# Delay along the care-seeking journey of patients with ocular surface squamous neoplasia in Kenya

**DOI:** 10.1186/s12913-017-2428-4

**Published:** 2017-07-14

**Authors:** Stephen Gichuhi, Joy Kabiru, Alain M’bongo Zindamoyen, Hillary Rono, Ernest Ollando, Joseph Wachira, Rhoda Munene, Timothy Onyuma, Mandeep S. Sagoo, David Macleod, Helen A. Weiss, Matthew J. Burton

**Affiliations:** 1 International Centre for Eye Health, London School of Hygiene and Tropical Medicine, Keppel Street, London, WC1E 7HT UK; 2 Department of Ophthalmology, University of Nairobi, P.O Box 19676-00202, Nairobi, Kenya; 3 PCEA Kikuyu Eye Unit, PO Box 45, Kikuyu, Kenya; 4 Kitale District Hospital, PO Box 98-30200, Kitale, Kenya; 5 Sabatia Eye Hospital, PO Box 214-50311, Wodanga, Kenya; 6 Kenyatta National Hospital, PO Box 20723-00202, Nairobi, Kenya; 7 Department of Pathology, MP Shah Hospital, PO Box 14497-00800, Nairobi, Kenya; 8 UCL Institute of Ophthalmology, 11-43 Bath Street, London, EC1V 9EL UK; 9 Moorfields Eye Hospital, 162 City Road, London, EC1V 2PD UK; 10 St. Bartholomew’s Hospital, W Smithfield, London, London EC1A 7BE UK; 11 MRC Tropical Epidemiology Group, London School of Hygiene and Tropical Medicine, Keppel Street, London, WC1E 7HT UK

**Keywords:** Ocular surface squamous neoplasia, Delay, Eye health, Health system, Referral, Women, women’s health, Care-seeking

## Abstract

**Background:**

In Africa, accessing eye health services is a major challenge. Ocular surface squamous neoplasia (OSSN) is a substantial ocular health problem in Africa related to solar UV light exposure and HIV infection among other risk factors. The disease causes visual loss and even death in advanced cases. This study was conducted to assess referral pathway and treatment delay for patients with OSSN in Kenya.

**Methods:**

Adults with conjunctival lesions presenting to four eye centres were asked about their occupations, when they noticed the growth, health facilities visited in seeking care, cost of consultation, surgery, medicines and histopathology and dates at each step. The time-to-presentation was divided into quartiles and correlates analysed using ordinal logistic regression.

**Results:**

We evaluated 158 first-time presenters with OSSN. Most were women (102 [65%]), living with HIV (78/110 tested [71%]), with low to medium income (127 [80%]). Most of the HIV patients (49/78 [63%]) were in antiretroviral care programs. About half (88/158, [56%]) presented directly to the study centres while the rest were referred. Indirect presenters sought care earlier than direct presenters (median 2.0 months vs 5.5 months) and travelled a shorter distance to the first health facility (median 20 km vs 30 km) but had surgery later (median 12.5 months vs 5.5 months). Visits beyond the first health facility for indirect presenters markedly increased delay (median 7.3, 29.0, 37.9, and 32.0 months for 1–4 facilities, respectively). Delay was associated with number of health facilities visited (adjusted ordered OR = 9.12; 95%CI 2.83–29.4, *p* < 0.001) and being female (adjusted ordered OR = 2.42; 95%CI 1.32–4.44, *p* = 0.004). At the time of presentation at the study centres for surgery the median tumour diameter in both directly and indirectly presenting patients was 6 mm (*p* = 0.52) and the histological spectrum of OSSN was similar between the groups (*p* = 0.87).

**Conclusions:**

Referral delays definitive treatment for OSSN. Women were more likely to experience delay. Despite regular contact with the health system for those with known HIV infection, delays occurred. Early detection and referral of OSSN in the HIV service might reduce delays, but reassuringly delay did not give rise to a larger proportion with more advanced grade of OSSN.

**Electronic supplementary material:**

The online version of this article (doi:10.1186/s12913-017-2428-4) contains supplementary material, which is available to authorized users.

## Background

Ocular surface squamous neoplasia (OSSN) usually presents as a defined lesion of the conjunctiva and/or cornea. In temperate countries it presents as a rare, slow-growing tumour mostly affecting elderly men. In contrast, in sub-Saharan Africa (SSA) OSSN is more common than in other global regions (1.38 and 1.18 cases/100000/year in males and females respectively in Africa vs 0.18 and 0.08 cases/100,000/year in males and females respectively worldwide). It is also more aggressive in SSA, predominantly affects people living with HIV (PLWH), and occurs with similar frequency in men and women [1, 2]. A study from Kenya found that people with no formal education were at higher risk of OSSN and presented with larger tumours than more educated individuals [3]. Late presentation with large tumours and orbital spread is not uncommon in Africa [4–6]. Surgery is the mainstay of treatment and if it is provided in a timely manner, outcomes can be very good with very low recurrence rates [7].

The Kenya health care referral system is organized into six levels [8]. The first level comprises community units. Services here focus on health promotion and treatment of minor ailments. Dispensaries form the second level. The third level comprises health centres with basic outpatient care, minor surgical services, basic laboratory services, maternity care, and limited inpatient facilities. They also coordinate the community units under their jurisdiction. Levels 4 and 5 are secondary-care hospitals providing curative services, and some training centres. Level 6 are tertiary referral facilities that offer specialised care and training to health workers. There are also a number of private and mission hospitals.

In Africa, accessing eye health services is a major challenge. A study in the Kibera slums and Dagoretti area of Nairobi found that the main barriers to utilization of eye health services were a lack of a perceived need for treatment (49% of respondents), lack of money (33% of respondents), while a small proportion (8%) did not know where to obtain help [9]. A study from Tanzania found that for eye trauma cases an injury at the weekend, use of topical treatment, and visiting other facilities were independently associated with delay of more than 24 h in accessing specialist eye care. Circular journeys were common where patients repeatedly visited health facilities that were unable to treat the injury [10].

Cancer is a growing problem in Africa. The age-standardised incidence rates for most cancers increased by more than 10% in most African countries between 1990 and 2013 [11]. A literature review of current cancer prevention approaches revealed that the major impediments to good care include a lack of awareness, limited human and financial resources, limited vaccine use (with regard to human papilloma virus), inadequacy of cancer registries, fear, cultural reasons (such as examination by doctors of the opposite sex) and competing health demands [12]. A study from Rwanda examined health system delays in breast cancer presentation and diagnosis of more than 6 months and found that low levels of education and consulting a traditional healer before a nurse or doctor were the main predictors of delay after adjusting for sociodemographic and clinical characteristics [13]. Visiting 5 or more health facilities before the diagnosis was associated with even longer delay in that study. The experiences of breast cancer, Kaposi’s sarcoma and lymphoma patients in the only oncology centre in Cameroon show that 35% of patients waited >6 months after the first sign of disease before presenting to the health system, while diagnosis was made >3 months after presentation in 47% of cases [14]. In the Cameroon study the total delay between first sign of cancer and a correct diagnosis was >6 months for 63% of patients. There is limited data on access to eye cancer services. This study was conducted to describe the presentation and referral “journey” that OSSN patients travel in Kenya, describing the time spent, identifying the points of delay and determining the factors associated with delay.

## Methods

This study was conducted in Kenya between July 2012 and July 2014. We used Ministry of Health records to identify four eye centres that performed the highest number of conjunctival excision biopsies between 2008 and 2011. The centres were Kenyatta National Hospital in Nairobi, PCEA Kikuyu Eye Unit (25 km from Nairobi in Central Kenya), Kitale district hospital in the north Rift Valley (490 km from Nairobi) and Sabatia Eye Hospital (300 km from Nairobi) in the western highlands bordering Lake Victoria. These centres receive referrals from surrounding health facilities.

We prospectively recruited consecutive adult patients (≥18 years) presenting to the four centres with any conjunctival lesion (first presentation or recurrence) suspected to be OSSN and scheduled for surgical excision. A detailed history was taken using a structured questionnaire before surgery. Participants were asked when they had first noticed the ocular lesion. To document the referral route taken prior to presentation, participants were asked to list the health facilities they had visited, their location, dates visited, advice given and total cost of clinical care to the patient (consultation, tests, surgery and medications). The first facility the patient visited was denoted as Facility 1, the second one visited (either as a result of formal referral or self-initiated referral) was denoted Facility 2 and so on. A comprehensive clinical examination was conducted at the slit lamp, including measurement of the longest lesion diameter using the slit lamp beam and scale. Participants underwent surgical excision under local anaesthesia. A histopathologist examined all the tissue specimens at MP Shah Hospital laboratory, Nairobi. Individuals who had histologically confirmed OSSN were offered a HIV test; if this was positive they were offered a referral for care and a CD4 count test. The detailed description of clinical assessment, surgery, tissue handling and testing have been previously published [3].

Treatment “delay” was defined as the time between awareness of having tumour lesion and having surgery at the study centre. For the purpose of this analysis we only included individuals who had histologically confirmed OSSN. As recurrence after a prior excision is likely to influence health-seeking behaviour and the journey followed, we excluded those with recurrent lesions from this analysis.

Data on the other health facilities visited by patients prior to presentation to one of the four study centres (location, health system level, ownership etc.) were obtained from the Kenya Ministry of Health web-based master facility list (http://kmhfl.health.go.ke/#/home) accessed on 15th November 2015. Information about the availability of ophthalmic surgical facilities (eye operating theatre) at each of these other health facilities was obtained from the Kenya Ministry of Health, Department of Ophthalmic Services (DOS). One-way distances travelled by road from the patients’ home town to the various health facilities and study centres were estimated using Google Maps following the roads that public vehicles would use.

We compared patients who presented directly to one of the four centres (“direct presenters”) with those who had visited other health facilities prior to presenting to a study centre (“indirect presenters”). Characteristics of the two groups were compared using the chi-squared test for proportions, and the t-test or Mann-Whitney U-test as appropriate for continuous variables. We traced the care seeking journey to describe the time taken, and the advice given at each stage. To investigate factors associated with delayed presentation we subdivided the total time in the range from awareness to surgery into quartiles and created an ordered categorical outcome variable. Ordinal logistic regression was used to estimate odds ratios (OR) and 95% confidence intervals (95%CI).

The likelihood ratio test was used to assess which factors from the univariable analysis would be included in the multivariable regression model. Variables that were associated with delay at a level of *p* < 0.1 in the univariable model were included in an initial multivariable model. The final multivariable model included variables which were independently associated with the outcome (*p* < 0.05). All statistical analyses were performed using Stata version 12.1 (StataCorp, College Station, Texas, USA).

## Results

A total of 500 individuals with conjunctival lesions were enrolled in this study and underwent surgical excision. Of these, 191 (38.2%) were diagnosed with OSSN by histopathology. Among these, 158 (83%) had new lesions and 33 (17%) had recurrent lesions (at least one prior excision at the same site).

**Fig. 1:**
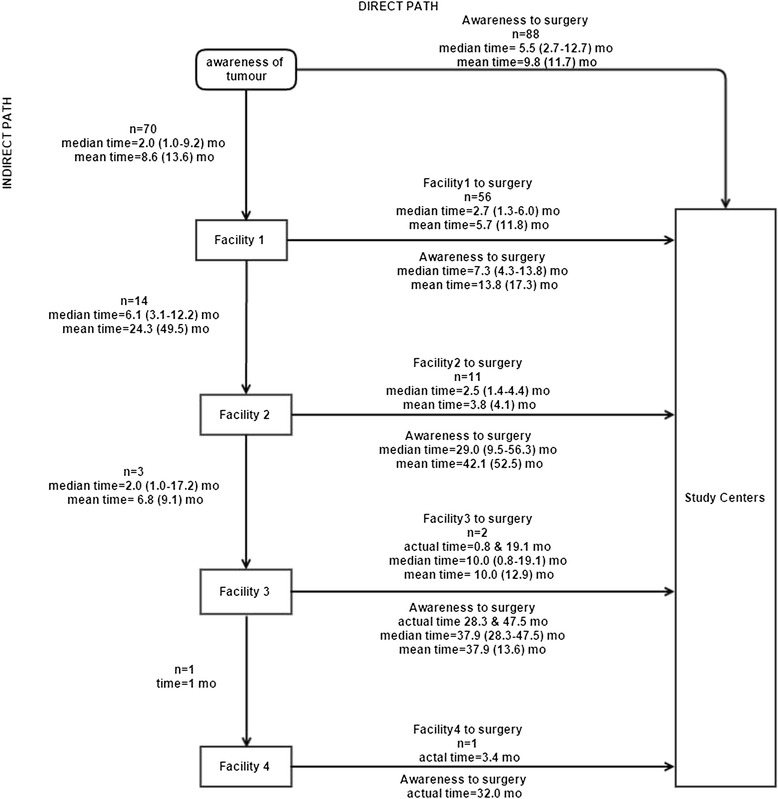
The care-seeking journey followed by 158 new OSSN patients and the duration of each step

The care-seeking journey of the 158 patients with new lesions was analysed: 88 (55.7%) presented directly to study centres and 70 (44.3%) presented indirectly via one or more other health facilities (Fig. 1). Overall, the mean age was 41.9 years (SD 12.0) and the majority were females (102 [65%]). Education levels were variable, 125 (79%) had completed primary school or above, and 67 (42%) had completed secondary school. The majority worked mostly outdoors (101 [65%]). Most occupations were low or middle income in nature (127 [80%]) and were predominantly in the agricultural sector. HIV status and CD4 count were obtained for patients who returned after surgery (110 [70%]. Of these, 78 (71%) were HIV positive, of whom 49 (63%) were already on antiretroviral therapy (ART).

The numbers of individuals taking different routes to the study centres and the time spent along each stage on the care-seeking journey is illustrated in Fig. 1. Most indirect presenters (56, [80%]) visited one other health facility before going to the study centres. Overall, despite having a shorter time to first presentation, indirect presenters took longer to receive surgery than direct presenters (5.5 vs 9.6 months, *p* = 0.001). The additional time in getting from the first health facility to the study centre was largely responsible for this difference. The longest delay in the whole care pathway occurred when patients went from the first facility to a second one (median 6.1 months, IQR 3.1–12.2). Referral beyond the first facility markedly increased delay.

The demographic and clinical characteristics of participants, by type of presentation, are shown in Table 1. There was little evidence of a difference between direct and indirect presenters by age, marital status, education, occupation, past experience of receiving eyecare or HIV status. There was weak evidence that indirect presenters were more likely to be female than direct presenters (71% vs 59%; *p* = 0.11). There was evidence that the main presenting symptom was different (*p* = 0.03) between the two groups, with pain more common in the direct presenters (17.1% vs 2.9%). At the time of presentation at the study centres for surgery the median tumour diameter in both directly and indirectly presenting patients was 6 mm (*p* = 0.52) and the histological spectrum of OSSN was similar between the groups (*p* = 0.87). Kikuyu Eye Unit and Kenyatta National Hospital had a significantly larger proportion of referrals than the other two study centres (*p* < 0.001). Direct presenters travelled a longer distance to the first health facility (study centre) than indirect presenters (median 30 km vs 20 km, *p* = 0.003).

**Table 1:** Demographic and clinical characteristics of 158 OSSN patients presenting directly or indirectly to study centres

Demographic or clinical feature	Direct presenters *N* = 88		Indirect presenters *N* = 70		*p*-value^a^
	n	(%)	n	(%)	
Age in years, mean (SD), y	42.5	(12.0)	41.1	(12.0)	0.47^c^
Sex, No. (%)					0.11
Male	36	(40.9)	20	(28.6)	
Female	52	(59.1)	50	(71.4)	
Marital status, No. (%)					0.21
Single	16	(18.2)	9	(12.9)	
Married	55	(62.5)	49	(70.0)	
Divorced or Separated	3	(3.4)	6	(8.6)	
Widowed	14	(15.9)	6	(8.6)	
Highest education level, No. (%)					0.25
Completed secondary or higher	33	(37.5)	34	(48.6)	
Completed primary or some secondary	33	(37.5)	25	(35.7)	
None or some primary	22	(25.0)	11	(15.7)	
Location of primary occupation, No. (%)					0.66
Indoor	29	(33.0)	26	(37.1)	
Outdoor	57	(64.8)	44	(62.9)	
Missing data	2	(2.2)	0	0	
Employment					0.23
Unemployed/no regular income	5	(5.7)	8	(11.4)	
Low to middle income	69	(78.4)	58	(82.9)	
High income	10	(11.4)	4	(5.7)	
Missing data	4	(4.6)	0	0	
HIV infection/ART use, No. (%)					0.42
HIV-	15	(25.0)	17	(34.0)	
HIV+/ART-	15	(25.0)	14	(28.0)	
HIV+/ART+	30	(50.0)	19	(380)	
CD4 count in cells/mm^3^, median(IQR)	344	(148–802)	219	(120–670)	0.42^d^
HIV-associated immunodeficiency by CD4 count in cells/mm^3^, No. (%)					0.28
None or not significant (≥500)	15	(37.5)	14	(34.2)	
Mild (350–499)	5	(12.5)	3	(7.3)	
Advanced (200–349)	9	(22.5)	5	(12.2)	
Severe (<200)	11	(27.5)	19	(46.3)	
Main symptom, No. (%)					0.03
Lump	53	(60.2)	48	(68.6)	
Pain	15	(17.1)	2	(2.9)	
Redness	6	(6.8)	8	(11.4)	
Others	14	(15.9)	12	(17.1)	
Tumour diameter in mm, median(IQR)	6.0	(4.3–8.5)	6.0	(4.2–10.0)	0.52^d^
Histopathology, No. (%)					0.87
CIN I (mild dysplasia)	5	(5.7)	4	(5.7)	
CIN II (moderate dysplasia)	13	(14.8)	9	(12.9)	
CIN III (severe dysplasia)	19	(21.6)	13	(18.6)	
Carcinoma-in-situ	0	0	1	(1.4)	
SCC – poorly differentiated	1	(1.4)	1	(1.4)	
SCC – moderately differentiated	45	(51.1)	35	(50.0)	
SCC – well differentiated	5	(5.7)	7	(10.0)	
Study Centre, No. (%)					<0.001
Kikuyu Eye Unit	42	(47.7)	55	(78.6)	
Kenyatta National Hospital	4	(4.6)	9	(12.9)	
Sabatia Eye Hospital	25	(28.4)	3	(4.3)	
Kitale District Hospital	17	(19.3)	3	(4.3)	
Distance from home to study centre or to 1st health facility in km, median(IQR)^b^	30	(20–89)	20	(5–56)	0.003 ^d^
Cost of care in KSh, median (IQR)	3800	(3800–4800)	3880	(3800–4100)	0.01^d^

Abbreviations: ART – antiretroviral therapy; CIN – conjunctival intraepithelial neoplasia; SCC- squamous cell carcinoma; KSh – Kenyan shillings

^a^testing whether the distribution of each demographic feature is the same in direct and indirect presenters

^b^1 patient had missing data on distance, 48 missing data on HIV and 77 on CD4 count

^c^t-test with unequal variances

^d^Mann-Whitney U-test

The median cost of care was similar in the two groups at 3800 (IQR 3800–4800) Kenyan Shillings (KSh) for direct presenters and 3880 (IQR 3800–4100) KSh for indirect presenters. The exchange rate in May 2016 was 100 KSh to 1US$. Costs incurred along the indirect route were mainly a subsidised consultation fee charged in government hospitals as most of the participants studied did not receive surgery there plus the cost at the study centre. Costs at the study centres (including consultation, surgery, post-operative medication and histopathology) were: Kikuyu Eye Unit 3800KSh, Kenyatta National Hospital 4000KSh, Sabatia 4800 KSh and Kitale 3500 Ksh. All histology was done at MP Shah Hospital to reduce inter-observer variation in the diagnosis. Tissue specimens from Sabatia and Kitale hospitals were sent to Nairobi for histopathology using a courier service which added to the costs.

The level of the health facility and the availability of an operating theatre at the place where patients presenting indirectly first went to are shown in Table 2. For 24 patients it was not possible to determine whether surgical facilities were available, mostly because the patient could not correctly name the facility.

**Table 2:** Types of health facilities represented by Facility 1 in the care-seeking journey for indirect presenters

Health facility	Operating theatre available				TOTAL
	None	General theatre	Eye unit theatre	Unknown	n (%)
Dispensary or Health Centre	1	0	0	5	6 (8.6)
District or sub-district hospital	0	3	-	2	5 (7.1)
County referral hospital	0	5	17	0	22 (31.4)
Private clinic	2	0	8	3	13 (18.6)
Mission hospital	0	1	3	2	6 (8.6)
Outreach eye camp	6	0	0	0	6 (8.6)
Facility not identified	0	0	0	12	12 (17.1)
TOTAL, N (%)	9 (12.9)	9 (12.9)	28 (40.0)	24 (34.3)	70 (100.0)

NOTE: This shows where indirect presenters first entered the health system and the availability of operating theatres in those clinics. General theatre refers to an operating theatre that is shared by all departments. Eye unit theatre means the eye unit has its own operating theatre

The most common points of entry into the health system for indirect presenters were at level 5 county referral hospitals (22 [31.4%]) and private clinics (13 [18.6%]). It is noteworthy that for the majority of facilities an operating theatre was available (37 [53%]). All four of the study centres have a dedicated ophthalmic operating theatre.

The advice given to indirectly presenting patients at the other facilities is shown in Table 3.

**Table 3:** Advice given at each health facility along the indirect care-seeking journey

Step in journey	Advice given			Total patients seen
	Follow up n(%)	Surgery Offered n(%)	Referred n(%)	
Facility 1	31 (44.3)	5 (7.1)	32 (45.7)	70 ^a^
Theatre available	13	4	18	37
No theatre	1	0	2	3
missing information	17	1	12	30
Facility 2	3 (21.4)	2 (14.3)	9 (64.3)	14
Theatre available	1	1	7	9
No theatre	0	0	1	1
missing information	2	1	1	4
Facility 3	1 (33.3)	1 (33.3)	1 (33.3)	3
Theatre available	0	0	1	1
No theatre	0	0	0	0
missing information	1	1	0	2
Facility 4	0	0	1 (100)	1
Theatre available	0	0	0	0
No theatre	0	0	0	0
missing information	0	0	1	1

^a^Data on advice given was only available for 70 patients. There was missing data on the advice given to 2 patients at the first facility

At each point patients were referred, advised to re-attend for follow up or advised to have surgery. It is not clear why some health facilities with operating theatres did not offer surgery. Regardless of the advice, all patients eventually went to the study centres at various time points. From the first facility visited, 32 patients were formally referred to a study centre. Of these, 29 (91%) followed this advice and went directly to a study centre within a median of 2.3 months (IQR 1.2–3.9), while three patients went via other clinics first and reached a study centre at 1, 3 and 42 months. Out of the 31 advised to re-attend for follow up, only one returned to the same facility seven months later and was then referred to one of the study centres for surgery. The other 30 either self-referred to a study centre (20 [65%]) and received surgery with a median time of 6.1 months (IQR 3.0–11.9) or to another clinic (10 [32%]). The five who were advised to have surgery all went to the study centres within a median (IQR) duration of 5.6 (2.3–11.5) months.

**Fig. 2:**
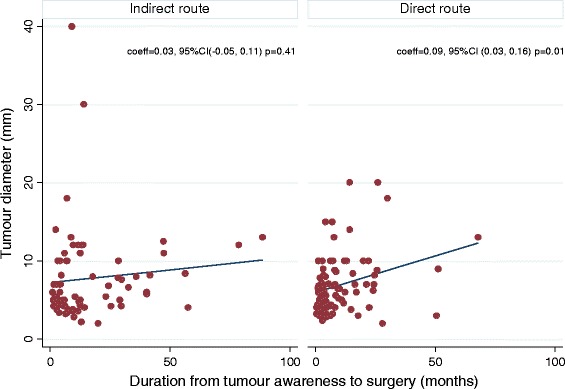
Scatterplots showing tumour size against delay between tumour awareness and having surgery at study centres

There was a modest linear relationship between duration of symptoms and the lesion size (Fig. 2) for direct presenters (*p* = 0.01), but not indirect presenters (*p* = 0.41). For the direct presenters, those who presented later tended to have a larger lesion, with an expected increase in size of 0.09 mm per month (95% CI 0.03–0.16). There is little evidence that the relationship between symptom duration and tumour size was different in direct and indirect presenters (*p* = 0.33). Delay was associated with larger tumour size at presentation (OR 1.07, 95%CI 1.01–1.13, *p* = 0.02). The median (IQR) tumour diameter was 5.6 (4.2–7.0), 5.0 (4.2–7.1), 6.4 (4.2–11.5) and 7.6 (5.4–10.0) millimetres (mm) in the following delay categories; 0.4–3.2, 3.3–7.0, 7.1–15.6 and 15.7–190.5 months respectively. Of the 158, 15 developed recurrences at one year, 7/88 (8.0%) from the direct group and 8/70 (11.4%) from the indirect group (*p* = 0.46). The recurrence rate was therefore not worse in either group. A sub-group analysis of indirect presenters comparing those who visited one facility to those who visited two or more facilities before the study centres did not show any significant differences (Additional file 1: Table S1).

Analysis of the predictors of delay is shown in Table 4. On multivariable ordered logistic regression, the number of health facilities visited in the care pathway followed and being female are associated with increased delay. Participants who followed an indirect path were twice as likely to be in a higher delay group than those in the direct pathway (adjusted OR = 2.3, 95%CI 1.5–3.7; *p* < 0.001). Similarly, females were twice as likely to be in a higher delay group than males (adjusted OR = 2.1; 95% CI 1.15–3.81, *p* = 0.02).

**Table 4:** Predictors of delay in presentation of 158 new patients with OSSN, sub-divided into quartiles

Factor	0.4–3.2 months (*N* = 38)	3.3–7.0 months (*N* = 41)	7.1–15.6 months (*N* = 40)	15.7–190.5 months (*N* = 39)	Univariate ordered proportional OR (95% CI)	*p-* value	Adjusted ordered proportional OR (95% CI)	*p-* value
Distance from home (in 10 km units) to first health facility or study center, mean(SD)	7.1 (10.2)	6.3 (8.2)	4.3 (5.0)	6.6 (9.6)	0.99 (0.95–1.02)	0.53	−	
Total cost of care (in 100Ksh units), mean (SD)	41.0 (6.0)	40.0 (5.0)	42.2 (15.9)	42.9 (8.4)	1.01 (0.99–1.04)	0.32		
Care pathway followed, No (%)						<0.001		<0.001
Direct	27 (30.7)	25 (28.4)	19 (21.6)	17 (19.3)	1.00 (REF)		1.00 (REF))	
1 facility visited	11 (19.6)	15 (26.8)	18 (32.1)	12 (21.4)	1.56 (0.85–2.83)		1.38 (0.75–2.54)	
2 or more facilities visited	0	1 (7.1)	3 (21.4)	10 (74.1)	12.93 (3.79–44.06)		13.03 (3.78–44.94)	
Sex, No. (%)								0.01
Male	18 (32.1)	18 (32.1)	12 (21.4)	8 (14.3)	1.00 (REF)	−	1.00 (REF)	
Female	20 (19.6)	23 (23.6)	28 (27.5)	31 (30.4)	2.29 (1.27–4.15)	0.006	2.31 (1.25–4.24)	
Age, mean(SD) y	42 (13.0)	40 (11.8)	46 (13.6)	39 (8.3)	0.99 (0.97–1.02)	0.69	−	
Marital status, No. (%)						0.59	−	
Single	2 (8.0)	11 (44.4)	3 (12.0)	9 (36.0)	1.00 (REF)			
Married	28 (26.9)	24 (23.1)	31 (29.8)	21 (20.2)	0.60 (0.28–1.31)			
Divorced or Separated	1 (11.1)	5 (55.6)	1 (11.0)	2 (22.2)	061 (0.17–2.26)			
Widowed	7 (35.0)	1 (5.0)	5 (25.0)	7 (35.0)	0.81 (0.27–2.45)			
Highest education level, No. (%)						0.23	−	
Completed secondary or higher	14 (20.9)	19 (28.4)	15 (22.4)	19 (28.4)	1.00 (REF)			
Completed primary or some secondary	12 (20.7)	16 (27.6)	14 (24.1)	16 (27.6)	1.01 (0.54–1.89)			
None or some primary	12 (36.4)	6 (18.2)	11 (33.3)	4 (12.1)	0.55 (0.26–1.17)			
Occupation, No. (%)						0.95	−	
High income	3 (21.4)	3 (21.4)	6 (42.9)	2 (14.3)	1.00 (REF)			
Low-Medium income	31 (24.4)	35 (27.6)	30 (23.6)	31 (234)	0.96 (0.37–2.48)			
Unemployed	4 (30.8)	3 (23.1)	3 (23.1)	3 (23.1)	0.82 (0.21–3.12)			
HIV infection & ART use, No. (%)^a^						0.07	−	
HIV-	11 (34.4)	10 (31.3)	6 (18.8)	5 (15.6)	1.00 (REF)			
HIV+/ART-	6 (20.7)	11 (37.9)	8 (27.6)	4 (13.8)	1.41 (0.58–3.40)			
HIV+/ART+	11 (22.5)	9 (18.4)	12 (24.5)	17 (34.7)	2.57 (1.13–5.88)			
Main symptom, No. (%)					1.07 (0.94–1.21)	0.32		
Lump	24 (23.8)	24 (23.8)	29 (28.7)	24 (23.8)				
Pain	7 (41.2)	5 (29.4)	2 (11.8)	3 (17.7)				
Redness	4 (28.6)	4 (28.6)	2 (14.3)	4 (28.6)				
Others	3 (11.5)	8 (30.8)	7 (26.9)	8 (308)				

^a^ There were 48 participants with missing HIV/ART data

## Discussion

This study identified various challenges along the journey to treatment for new patients with OSSN. Firstly, referral significantly delayed surgery and delay was associated with larger tumours at the time of surgery. Patients in the indirect route initially presented earlier than those who went directly to study centres perhaps because it was a shorter distance and the cost of care was lower. However, surgery was more delayed in the indirect route compared to the direct (5.5 months vs 9.6 months, *p* = 0.001), particularly if the patients visited more than one health facility. This correlates well with the finding that the number of facilities visited before was an important predictor of delay in receiving surgery. When definitive treatment was carried out in the study centres, there was no significant difference in the tumour size nor the histological spectrum of OSSN was similar between the direct early presenting and the indirect delayed presenting groups (*p* = 0.87).

Secondly, we found being female was another important predictor of delay. Gender disparities in access to health care services have also been identified in East Africa for other ocular diseases such as eye trauma and cataract with females having relatively more difficulty [10, 15]. A study on access to cataract surgery in Tanzania found that some women needed to seek permission from their husbands before going to hospital or may rather put up with the adversity of poor eye health for fear of being seen as a burden in the family [15]. Women are less likely than men to be the financial decision makers with regard to seeking health care in this population. In addition, they may have other responsibilities to consider before going to hospital such as child care and home upkeep. Although there is limited information on gender-specific utilization of cancer services in Africa, particularly for OSSN, we hypothesize that women with household and child-care responsibilities have more difficulty attending health facilities, particularly if distant referrals are made.

With respect to the patients reported advice and treatment provided at facilities other than the four study centres there is an intrinsic limitation in that we do not know how many other (if any) people had excisions in the other health facilities who never came to one of the study centres. There is no reporting of this to the central Ministry of Health. However, it would appear that advising follow up for suspicious lesions needs to be supported by more than just a clinical impression. At the first health facility level, 20 of 31 patients who were advised to return for follow up went to the study centres and had surgery within median 6 months (mean 13 months) and were found to have OSSN (Table 3). Only one patient at the first facility took the follow up advice and by the time of review seven months later was referred for surgery and was found to have OSSN. While we have no information on how the lesions looked at earlier time points, they may have appeared either benign or were suspicious of malignancy and progressed rather rapidly within a few months, underscoring two things, that OSSN in East Africa is an aggressive disease and that distinguishing OSSN from benign lesions clinically is challenging as they can look very similar [3].

It is still unclear what drives the decision to present to a first health facility once the person become aware of the tumour. Perhaps it is more the absolute size rather than the rate of growth or combination of the two. We found only a modest difference in growth rates between direct and indirect presenters. It is likely that the duration and growth rates will vary. Pain maybe an important driver of the decision; we found that pain was more common among the direct presenters (*p* = 0.02).

The majority of participants were living with HIV and were already in contact with HIV care and treatment programs. Despite this, they still experienced delay. There could be various explanations for this. Firstly, awareness about OSSN among HIV health workers may be limited. Secondly, patients on ART may find it particularly challenging to seek care for OSSN. Over half (44/81 [54%]) the patients on ART had advanced or severe immunosuppression and thus at high risk for comorbidities such as tuberculosis [16]. Thirdly, due to stigma around HIV they may not feel safe or comfortable in other health facilities. A recent study in rural western Kenya found that cervical cancer stigma was highly correlated with HIV stigma (correlation coefficient 0.72) [17].

We did not find income or level of formal education a barrier to presentation despite most of our patients (80%) being in the low to medium income group. The majority worked in farming. Most employment (83%) nationwide is in the informal sector so it is difficult to know the real earnings as they are not captured in tax systems [18]. In Kenya half of healthcare expenditure (51.1%) is paid for out-of-pocket [19]. The gazetted monthly basic minimum wages for the agricultural industry during the study period was an average of KSh 5704 in 2012 rising to KSh 6503 in 2013 and remained the same in 2014 which was comparable to the fees charged at the study centres [20]. However the opportunity cost of lost income during the care-seeking period for the patient and other adults involved in supporting this care could not be estimated. A study in Kenya found that households spend over 10% of their annual income on healthcare payments with the poor spending more than 33% [21]. The mean annual total spending per household (both inpatient and outpatient) was KSh 16,000 (£114) in urban areas and KSh 5600 (£40) in rural areas. Only about 7% of Kenyans have private health insurance [20]. The National Health Insurance Fund (NHIF) run by the government is the dominant insurance provider. The maximum contribution was KSh 320 (about £2) per month until April 2015 when it was increased to KSh 1700 (£12) per month. NHIF used to pay for the inpatient bed charge only, therefore day surgery (the norm for OSSN surgery) would not be covered. This situation has now been reviewed. The cost of travel is not captured in this analysis. Usually most patients in Kenya would travel to hospitals accompanied by a relative or friend, further increasing the cost of care.

This study had several weaknesses. Data on tumour awareness, cost of care and referral details were obtained from a structured questionnaire before surgery rather than medical records from referring facilities. The questionnaire, while practical, may not be the most accurate tool to evaluate care-seeking behaviour. The responses were subject to recall bias. We also did not capture all past experiences in receiving eyecare to see if this affected care-seeking behaviour. The analysis did not address the reliability of the initial diagnosis in the indirect presenters nor the availability of eye surgeons or other surgeons skilled enough and adequately equipped with the right surgical instruments to excise conjunctival lesions as a cause of referral and hence delay. These data were not available to us. The presence or absence of an operating theatre is probably not the determining factor for referral - it is likely to be the availability of an appropriately skilled surgeon. Our analysis of the flow through the health system did not address why the patients did what they did. To answer this would require a qualitative study.

## Conclusions

Women and those who visited more health facilities were at increased risk of delay. We observed various health system delays. Referral beyond the first point of contact was a major cause of delay. Reassuringly delay did not give rise to a larger proportion with more advanced grade of OSSN. It is also unclear why patients who are in regular contact with the health system through HIV care programs have delayed presentation. There is need to evaluate if health education about OSSN and the ocular effects of HIV infection particularly among HIV care workers is a barrier to the provision of timely intervention. Systematic OSSN screening could be considered in HIV care programs. Patients with pain presented more rapidly. The cost to the patient of treating OSSN (excluding transport casts) was equivalent to a month’s wages. It has been suggested that cancer care programs could learn from HIV programs by mainstreaming cancer into the health system together with advocacy and improved health worker training [22].

## Additional file

Additional file 1: Table S1. Sub-group analysis among indirect presenters. (DOCX 18 kb)

## Abbreviations

ART: Antiretroviral therapy
HIV: Human immunodeficiency virus
IQR: Interquartile range
KSh: Kenyan shillings
NHIF: National Health Insurance Fund
OSSN: Ocular surface squamous neoplasia
SSA: Sub Saharan Africa
