# Supplementary material for: Delay along the care-seeking journey of patients with ocular surface squamous neoplasia in Kenya
Source: BMC Health Serv Res. 2017 Jul 14;17:485. doi: 10.1186/s12913-017-2428-4 (PMC5512725; doi:10.1186/s12913-017-2428-4)
Supplement: Additional file 1: Table S1. — Sub-group analysis among indirect presenters. (DOCX 18 kb) [file 12913_2017_2428_MOESM1_ESM.docx]

**Additional file 1: Table S1. Sub-group analysis among indirect presenters****.**

| **Demographic and clinical features** | **Indirect presenters to 1 facility**  **N=56** | | **Indirect presenters to ≥ 2 facilities N=14** | | **p-value** ^a^ |
| --- | --- | --- | --- | --- | --- |
|  | **n** | **(%)** | **n** | **(%)** |  |
| Age in years, men (SD), y | 41.2 | (13.2) | 40.5 | (5.8) | 0.74^b^ |
| Sex, No. (%) |  |  |  |  | 0.51 |
| Male | 15 | (26.8) | 5 | (35.7) |  |
| Female | 41 | (73.2) | 9 | (64.3) |  |
| Marital status, No. (%) |  |  |  |  | 0.37 |
| Single | 9 | (16.1) | 0 | 0 |  |
| Married | 38 | (67.9) | 11 | (78.6) |  |
| Divorced or Separated | 4 | (7.1) | 2 | (14.3) |  |
| Widowed | 5 | (8.9) | 1 | (7.1) |  |
| Highest education level, No. (%) |  |  |  |  | 0.08 |
| Completed secondary or higher | 28 | (50.0) | 6 | (42.9) |  |
| Completed primary or some secondary | 17 | (30.4) | 8 | (57.1) |  |
| None or some primary | 11 | (19.6) | 0 | 0 |  |
| Location of primary occupation, No. (%) |  |  |  |  | 0.17 |
| Indoor | 23 | (41.1) | 3 | (21.4) |  |
| Outdoor | 33 | (58.9) | 11 | (78.6) |  |
| Employment |  |  |  |  | 0.47 |
| Unemployed/no regular income | 7 | (12.5) | 1 | (7.1) |  |
| Low to middle income | 45 | (80.4) | 13 | (92.9) |  |
| High income | 4 | (7.1) | 0 |  |  |
| Previous eye operation | 1 | (1.8) | 1 | (7.1) | 0.28 |
| History of allergic conjunctivitis | 10 | (17.9) | 5 | (35.7) | 0.15 |
| HIV infection/ART use, No. (%) |  |  |  |  | 0.33 |
| HIV- | 16 | (28.6) | 1 | (7.1) |  |
| HIV+/ART- | 11 | (19.6) | 3 | (21.4) |  |
| HIV+/ART+ | 15 | (26.8) | 4 | (28.5) |  |
| missing data | 14 | (25.0) | 6 | (42.9) |  |
| CD4 count in cells/mm^3^, median(IQR) | 219 | (120-715) | 179 | (69-407) | 0.48^c^ |
| Main symptom, No. (%) |  |  |  |  | 0.80 |
| Lump | 38 | (67.9) | 10 | (71.4) |  |
| Pain | 2 | (3.6) | 0 | 0 |  |
| Redness | 7 | (12.5) | 1 | (7.1) |  |
| Others | 9 | (16.1) | 3 | (21.4) |  |
| Tumour diameter in mm, median(IQR) | 5.4 | (4.2-10.0) | 8.0 | (5.4-12.0) | 0.06 |
| Histopathology, No. (%) |  |  |  |  | 0.93 |
| CIN I (mild dysplasia) | 4 | (7.1) | 0 | 0 |  |
| CIN II (moderate dysplasia) | 7 | (12.5) | 2 | (14.3) |  |
| CIN III (severe dysplasia) | 10 | (17.9) | 3 | (21.4) |  |
| Carcinoma-in-situ | 1 | (1.8) | 0 | 0 |  |
| SCC – poorly differentiated | 1 | (1.8) | 0 | 0 |  |
| SCC – moderately differentiated | 27 | (48.2) | 8 | (57.1) |  |
| SCC – well differentiated | 6 | (10.7) | 1 | (7.1) |  |
| Study Centre, No. (%) |  |  |  |  | 0.18 |
| Kikuyu Eye Unit | 45 | (80.4) | 10 | (71.4) |  |
| Kenyatta National Hospital | 5 | (8.9) | 4 | (28.6) |  |
| Sabatia Eye Hospital | 3 | (5.4) | 0 | 0 |  |
| Kitale District Hospital | 3 | (5.4) | 0 | 0 |  |
| Distance from home to 1st health facility in km, median(IQR) | 20 | (5-53) | 20 | (4-70) | 0.73^c^ |
| Cost of care in KSh, median (IQR) | 3865 | (3800-4150) | 3900 | (3800-4000) | 0.84^c^ |

Abbreviations: ART – antiretroviral therapy; CIN – conjunctival intraepithelial neoplasia; SCC- squamous cell carcinoma

^a^ testing whether the distribution of each feature is the same in indirect presenters to 1 facility and indirect presenters to ≥2 facilities; ^b^ t-test with unequal variances; ^c^ Mann-Whitney U-test
